# Random Urinary Calcium/Creatinine Ratio as a Predictor of Dehydration in Hypercalcemic and Normocalcemic Patients

**DOI:** 10.3390/jcm15186975

**Published:** 2026-09-09

**Authors:** Rotem Tal-Ben Ishay, Yossi Asaf, Michal Canetti, Michael Edery, Shir Elimeleh, Ameen Masri, Grace Rabinowitz, Neta Silbermintz, Gabriel Zollmann, Haim Mayan

**Affiliations:** 1 Department of Internal Medicine E, Sheba Medical Center, Tel HaShomer, Ramat Gan 52621, Israel; yosef.asaf@sheba.health.gov.il (Y.A.); michal.canetti@sheba.health.gov.il (M.C.); michael.edery@sheba.health.gov.il (M.E.); shir.elimeleh@sheba.health.gov.il (S.E.); ameen.masri@sheba.health.gov.il (A.M.); grace.rabinowitz@sheba.health.gov.il (G.R.); neta.silbermintz@sheba.health.gov.il (N.S.); gabriel.zollmann@sheba.health.gov.il (G.Z.); haim.maayan@sheba.health.gov.il (H.M.); 2 Faculty of Medicine, Tel Aviv University, Tel Aviv 69978, Israel

**Keywords:** dehydration, hypercalcemia, hypocalciuria, urinary calcium-to-creatinine ratio, fractional excretion of calcium, parathyroid hormone

## Abstract

**Background/Objectives:** Dehydration is an under-recognized cause of hypercalcemia, and no validated bedside marker exists to distinguish it from other etiologies. We evaluated the urinary calcium-to-creatinine ratio (uCa/Cr) and the fractional excretion of calcium (FeCa) as predictors of dehydration across a spectrum of hypercalcemic and normocalcemic states. **Methods:** We conducted a retrospective cohort study of 96 patients admitted to an internal medicine ward with dehydration and/or hypercalcemia. Dehydration was identified using a combination of clinical criteria (presenting complaint, physical examination findings of volume depletion, and admitting physician assessment) and biochemical criteria (elevated plasma urea, creatinine, or sodium, or elevated urine osmolality). Patients were classified into three groups: non-dehydrated hypercalcemic (n = 37), dehydrated hypercalcemic (n = 24), and dehydrated normocalcemic (n = 35). Urinary analyses were performed on patients not receiving chronic diuretic therapy (n = 70 with complete data). Logistic regression, receiver operating characteristic (ROC) analysis, and CKD-stratified sensitivity analyses were performed. **Results:** uCa/Cr differed significantly across all three groups (medians 1.046, 0.484, and 0.056 mmol/mmol respectively; *p* < 0.001 for all pairwise comparisons). Hypocalciuria-defined as reduced urinary calcium excretion (uCa/Cr < 0.2 mmol/mmol)-was absent in non-dehydrated patients and present in 59.5% of dehydrated patients; this should not be confused with hypocalcemia (low serum calcium). ROC analysis yielded an AUC of 0.894 (95% CI: 0.819–0.969), significantly superior to FeCa (AUC 0.813; DeLong *p* = 0.012). At a threshold of 0.2 mmol/mmol, specificity and positive predictive value were both 100%. uCa/Cr remained independently associated with dehydration after adjustment for chronic kidney disease (CKD), age, and sex (OR 0.112, 95% CI: 0.030–0.307, *p* < 0.001). PTH suppression was significantly less frequent in dehydrated than non-dehydrated patients (24.4% vs. 51.5%, *p* = 0.018). **Conclusions:** Random uCa/Cr is a highly specific, CKD-independent predictor of dehydration. A uCa/Cr value below 0.2 mmol/mmol, particularly combined with non-suppressed PTH, should prompt consideration of dehydration as the primary cause of hypercalcemia.

## 1. Introduction

Hypercalcemia is a common finding in hospitalized patients, occurring in up to 15% of admissions, and is most frequently attributed to malignancy or primary hyperparathyroidism [1]. Dehydration is an additional but under-recognized cause, acting through hemoconcentration and enhanced tubular calcium reabsorption [2,3]. Although fluid resuscitation is the cornerstone treatment for both dehydration and hypercalcemia, dehydration is rarely considered the primary etiology when a patient presents with elevated serum calcium [4,5].

The case that motivated this study illustrates this diagnostic gap: a twenty-five-year-old male with Cannabis Hyperemesis Syndrome was admitted repeatedly with vomiting, severe dehydration, and hypercalcemia (serum calcium up to 15.5 mg/dL). His urinary calcium-to-creatinine ratio (uCa/Cr) was strikingly low at 0.05 mmol/mmol, and both serum calcium and uCa/Cr normalized within 24 h of intravenous fluid resuscitation, confirming dehydration as the primary driver.

Volume depletion promotes calcium reabsorption in the distal tubule via sodium-coupled mechanisms, reducing urinary calcium excretion and producing hypocalciuria [6]. Clinically, hypercalcemia is classified as mild (corrected serum calcium 10.5–12.0 mg/dL), moderate (12.0–14.0 mg/dL), or severe (>14.0 mg/dL); levels above 12 mg/dL are generally considered symptomatic and levels above 14 mg/dL constitute a medical emergency requiring urgent intervention [3]. The diagnostic use of uCa/Cr has been established in thiazide-induced hypocalciuria [7] and Gitelman syndrome [8], where a ratio below 0.2 mmol/mmol is the accepted threshold. Whether the same pattern applies to dehydration from other causes has not been systematically studied.

Several methodological challenges complicate this question: there is no universally accepted gold standard for dehydration in hospitalized patients; chronic kidney disease (CKD) alters urinary calcium handling and may confound urinary calcium-based markers [9]; and the overlap between dehydration and other causes of hypercalcemia must be carefully characterized.

We designed this retrospective cohort study to evaluate uCa/Cr and the fractional excretion of calcium (FeCa) as predictors of dehydration across a spectrum of clinical presentations, including patients with and without concurrent hypercalcemia, and to determine whether these markers remain informative after adjustment for CKD and other confounders.

## 2. Materials and Methods

### 2.1. Study Design and Population

**Figure 1:**
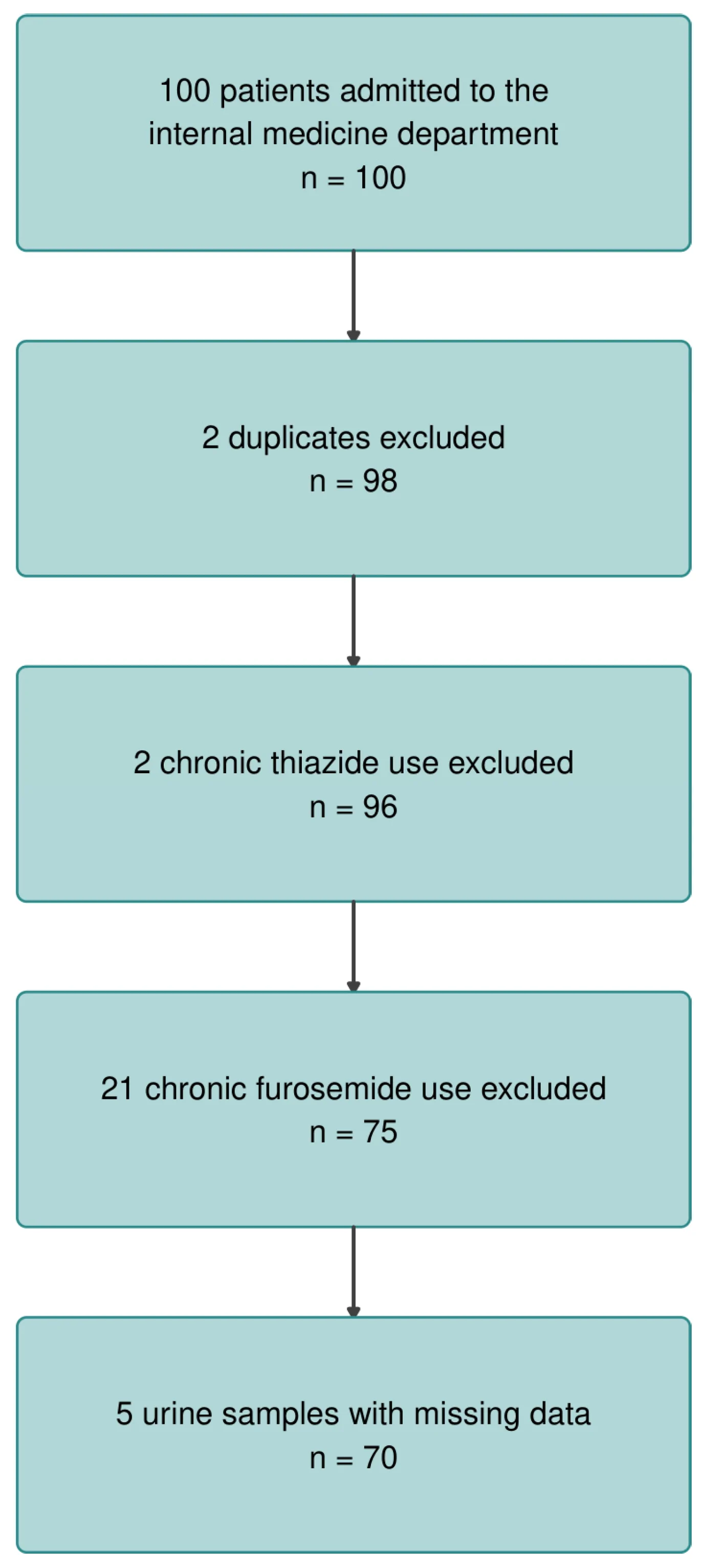
Patient flow diagram. Of 100 patients initially enrolled, 2 duplicate entries and 2 patients with chronic thiazide use were excluded, leaving 96 for primary analysis. Of 75 patients without chronic furosemide use, 5 had missing urine data, leaving 70 for urinary outcome analyses.

This is a retrospective cohort study of 100 patients admitted to the Department of Internal Medicine E, Sheba Medical Center, Israel, between 2020 and 2025, with a primary presentation of dehydration and/or hypercalcemia. Patient identifiers were recorded informally during routine clinical care between 2020 and 2024. Following formal ethics approval in February 2024, full data collection from medical records was completed and analysis was conducted in 2024–2025. Two duplicate entries were removed and two patients with chronic thiazide use were excluded, leaving 96 patients for primary analysis (Figure 1). The study was conducted in accordance with the Declaration of Helsinki and approved by the Helsinki Committee of Sheba Medical Center (protocol code SMC-0602-23, approved 6 March 2024, renewed 23 April 2026).

### 2.2. Patient Classification

Patients were classified into three groups based on clinical presentation, admission etiology, and biochemical findings: (1) non-dehydrated hypercalcemic patients (n = 37), with hypercalcemia attributed to malignancy, hyperparathyroidism, immobilization, iatrogenic causes, or unknown etiology; (2) dehydrated patients with concomitant hypercalcemia (n = 24); and (3) dehydrated patients without hypercalcemia (n = 35).

### 2.3. Dehydration Definition

Dehydration was defined as extracellular volume depletion, identified by a combination of clinical and biochemical criteria. Clinical criteria included the presenting complaint (vomiting, diarrhea, or poor oral intake), physical examination findings consistent with volume depletion (hypotension, dry mucous membranes, or reduced jugular venous pressure), and the admitting physician’s clinical assessment. Biochemical support was provided by at least one of the following: elevated plasma urea (>50 mg/dL), elevated creatinine (>1.2 mg/dL), hypernatremia (≥145 mEq/L), or elevated urine osmolality (>400 mOsm/kg). As a group, dehydrated patients had significantly higher plasma urea (median 105 vs. 49 mg/dL, *p* < 0.001), creatinine (1.72 vs. 0.90 mg/dL, *p* < 0.001), plasma sodium (141 vs. 137 mEq/L, *p* = 0.001), and urine osmolality (486 vs. 377 mOsm/kg, *p* = 0.015) compared to non-dehydrated patients.

### 2.4. Hypercalcemia Definition

Hypercalcemia was defined as an ionized calcium level above 1.32 mmol/L and a corrected calcium level above 10.5 mg/dL, consistent with current laboratory reference ranges [10]. Ionized calcium was measured directly using a blood gas analyzer (point-of-care testing); full acid–base parameters (pH, bicarbonate) were not systematically collected, and pH correction of ionized calcium was therefore not performed. Total calcium was corrected for albumin using the standard formula: corrected calcium (mg/dL) = measured calcium + 0.8 × (4.0 − albumin [g/dL]).

### 2.5. Data Collection

Data collected at admission included demographics, comorbidities (CKD, hypertension, diabetes mellitus, heart failure), chronic diuretic use, and serum biochemistry (sodium, potassium, urea, creatinine, albumin, total protein, total and corrected calcium, ionized calcium, parathyroid hormone [PTH], and 25-hydroxyvitamin D). Urine biochemistry collected concurrently included calcium, creatinine, sodium, urea, and osmolality. The uCa/Cr ratio was expressed in mmol/mmol. FeCa was calculated as follows: FeCa (%) = (urine calcium [mg/dL]/serum calcium [mg/dL])/(urine creatinine [mg/dL]/serum creatinine [mg/dL]) × 100. Hypocalciuria was defined as uCa/Cr < 0.2 mmol/mmol [8,11].

### 2.6. Diuretic Exclusion

Of the 96 patients, 21 (21.9%) were receiving chronic furosemide therapy. These patients were excluded from all urinary analyses due to the known effects of loop diuretics on urinary calcium and sodium excretion [12,13,14]. An additional five patients were excluded from urinary analyses due to missing urine samples, leaving 70 patients for urinary outcome analyses.

### 2.7. Statistical Analysis

Continuous variables are presented as median (interquartile range [IQR]). Categorical variables are presented as number and percentage. Between-group comparisons used the Kruskal–Wallis test with post hoc pairwise Mann–Whitney U tests (Bonferroni correction) for continuous variables, and the chi-square test for categorical variables. The association between uCa/Cr and dehydration was evaluated using logistic regression in three sequential models: uCa/Cr alone (Model 1); adjusted for CKD (Model 2); and additionally adjusted for age and sex (Model 3). Sensitivity, specificity, positive predictive value (PPV), and negative predictive value (NPV) were evaluated by receiver operating characteristic (ROC) analysis. AUC comparison between uCa/Cr and FeCa was performed using the DeLong method. Sensitivity analyses were conducted in CKD and non-CKD subgroups separately. Statistical analyses were performed using R version 4.6.0 (R Foundation for Statistical Computing, Vienna, Austria). A two-tailed *p*-value < 0.05 was considered statistically significant.

## 3. Results

### 3.1. Patient Characteristics

Ninety-six patients were included. Table 1 summarizes characteristics by group. The three groups did not differ significantly in age (*p* = 0.060), albumin (*p* = 0.301), total protein (*p* = 0.519), or sex distribution (*p* = 0.061). The dehydrated + hypercalcemic group had a notably higher proportion of females (67%) and a significantly higher prevalence of CKD (54.2%) compared to non-dehydrated (13.5%) and dehydrated + normocalcemic patients (25.7%, *p* = 0.001).

Both dehydrated groups had significantly higher plasma urea and creatinine than non-dehydrated patients (*p* < 0.001 for both). Plasma sodium was highest in the dehydrated + normocalcemic group (median 143 mEq/L), significantly exceeding non-dehydrated patients (137 mEq/L, *p* = 0.002). Although the fractional excretion of sodium (FeNa) and urea (FeUrea) did not differ significantly across groups (*p* = 0.634 and *p* = 0.170, respectively), median FeNa was directionally lower in dehydrated patients (0.7%) than non-dehydrated patients (1.1%). Urinary creatinine was significantly higher in both dehydrated groups compared to non-dehydrated patients (medians 92.8 and 83.6 vs. 40.7 mg/dL, *p* = 0.009), reflecting urine concentration.

### 3.2. PTH and Hypercalcemia Etiology

PTH data were available in 78 of 96 patients (18.8% missing). Among the 37 non-dehydrated patients, hypercalcemia etiologies were malignancy (n = 17, 45.9%), hyperparathyroidism (n = 12, 32.4%), immobilization (n = 3, 8.1%), unknown (n = 3, 8.1%), and iatrogenic (n = 1, 2.7%). PTH suppression (<20 pg/mL) was significantly more common in non-dehydrated hypercalcemic patients (51.5%) than in all dehydrated patients (24.4%, *p* = 0.018), consistent with non-PTH-mediated hypercalcemia predominating in the former group. PTH levels in the dehydrated + normocalcemic group were highest (median 57.2 pg/mL, IQR 40.5–92.0), suggesting secondary hyperparathyroidism driven by volume depletion.

### 3.3. UCa/Cr and FeCa Across Groups

**Figure 2:**
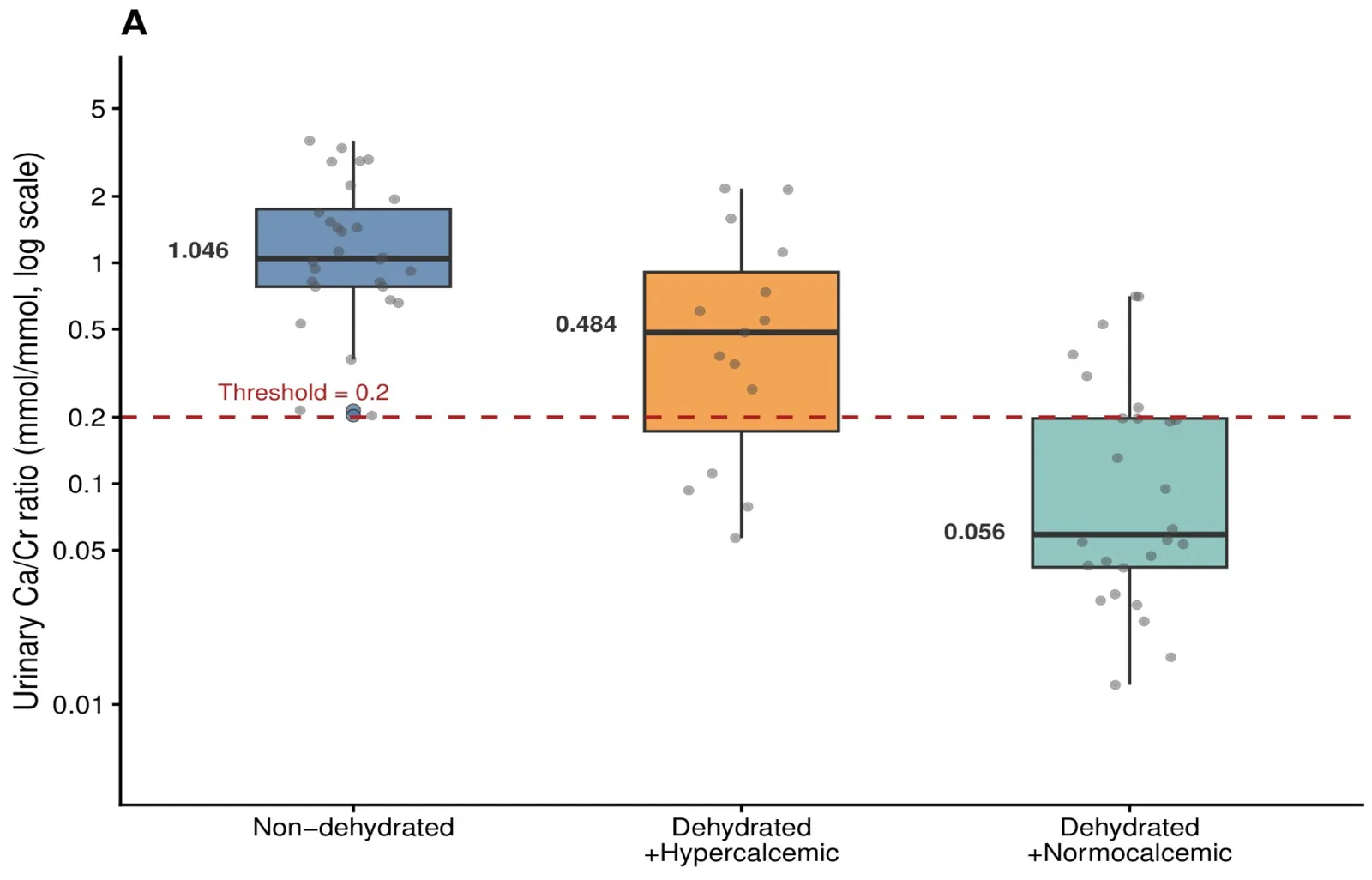

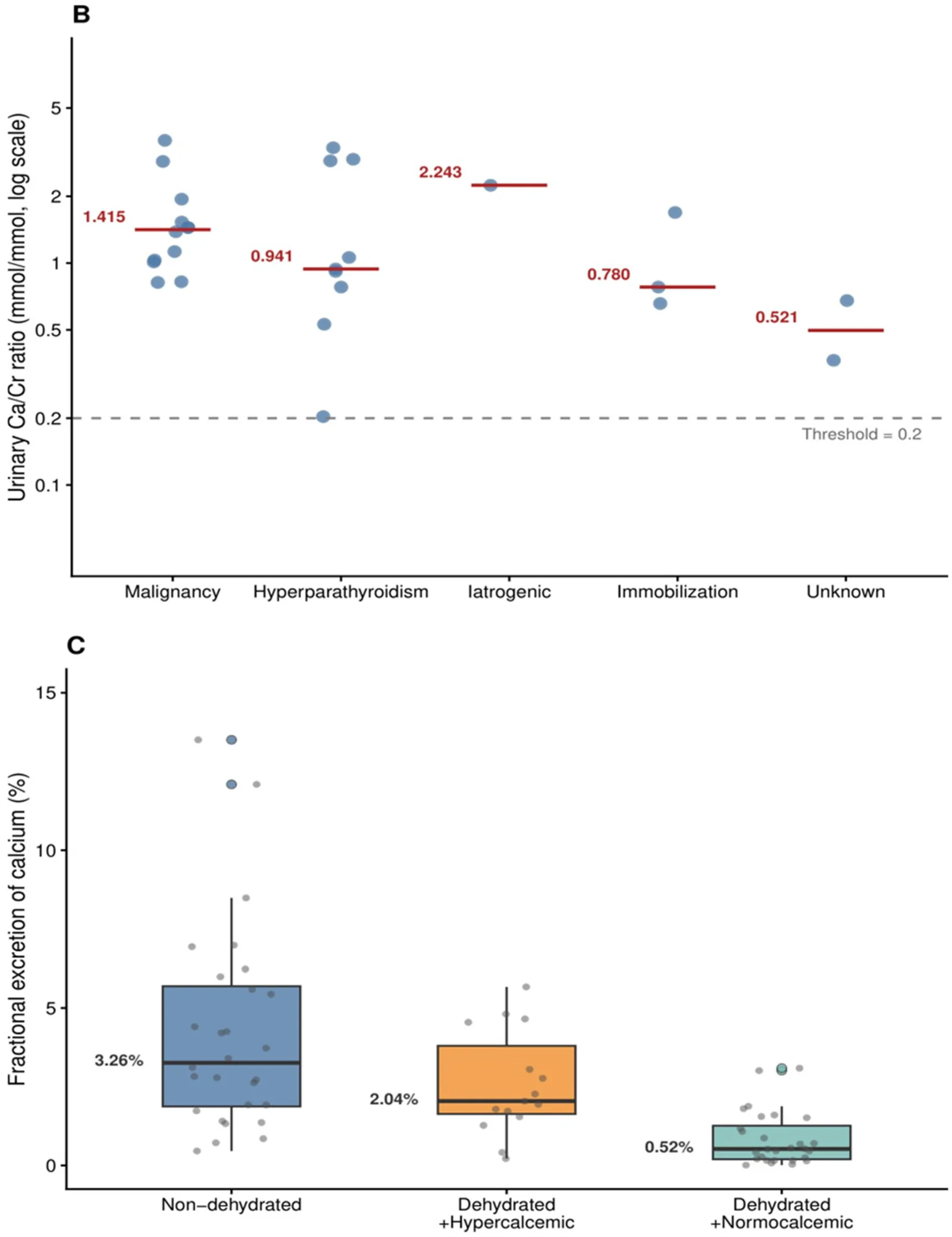
Urinary calcium-to-creatinine ratio (uCa/Cr) and fractional excretion of calcium (FeCa) by group and etiology. (**A**) uCa/Cr by patient group (n = 70, diuretic-filtered), log scale. Median values annotated. Dashed red line = hypocalciuria threshold (0.2 mmol/mmol). All pairwise comparisons were significant after Bonferroni correction (*p* ≤ 0.017). (**B**) uCa/Cr by hypercalcemia etiology in non-dehydrated patients. Dashed grey line = clinical cutoff of hypocalciuria (0.2). (**C**) FeCa by patient group. FeCa did not significantly distinguish non-dehydrated from dehydrated + hypercalcemic patients (Bonferroni *p* = 0.412), unlike uCa/Cr.

It is important to note that hypocalciuria refers to reduced urinary calcium excretion-a tubular phenomenon reflecting avid calcium conservation driven by volume depletion-and is entirely distinct from hypocalcemia (low serum calcium). All patients in this study had normal or elevated serum calcium; the finding of low urinary calcium excretion in dehydrated patients is mechanistically consistent with enhanced tubular reabsorption rather than any systemic calcium deficiency. Among the 70 patients with complete urine data and no chronic diuretic use, uCa/Cr differed significantly across all three groups (Kruskal–Wallis *p* < 0.001; Figure 2A). Median uCa/Cr was 1.046 mmol/mmol (IQR 0.780–1.754) in non-dehydrated patients, 0.484 mmol/mmol (IQR 0.189–0.927) in dehydrated + hypercalcemic patients, and 0.056 mmol/mmol (IQR 0.037–0.197) in dehydrated + normocalcemic patients. All pairwise comparisons were statistically significant after Bonferroni correction (non-dehydrated vs. dehydrated + hypercalcemic *p* = 0.017; non-dehydrated vs. dehydrated + normocalcemic *p* < 0.001; dehydrated + hypercalcemic vs. dehydrated + normocalcemic *p* = 0.001).

FeCa showed a similar stepwise pattern (medians 3.26%, 2.04%, and 0.52% respectively; Kruskal–Wallis *p* < 0.001; Figure 2C), but the distinction between non-dehydrated and dehydrated + hypercalcemic groups was not significant for FeCa (*p* = 0.412), whereas it was significant for uCa/Cr (*p* = 0.017). uCa/Cr and FeCa were strongly correlated (Spearman ρ = 0.883, *p* < 0.001). Among non-dehydrated patients, uCa/Cr was highest in malignancy (median 1.415) and hyperparathyroidism (median 0.941), and lowest in immobilization (0.780) and iatrogenic causes (0.521; Figure 2B).

Hypocalciuria (uCa/Cr < 0.2 mmol/mmol) was absent in all 28 non-dehydrated patients (0%) and present in 25 of 42 dehydrated patients (59.5%, *p* < 0.001).

### 3.4. Diagnostic Performance: ROC Analysis

**Figure 3:**
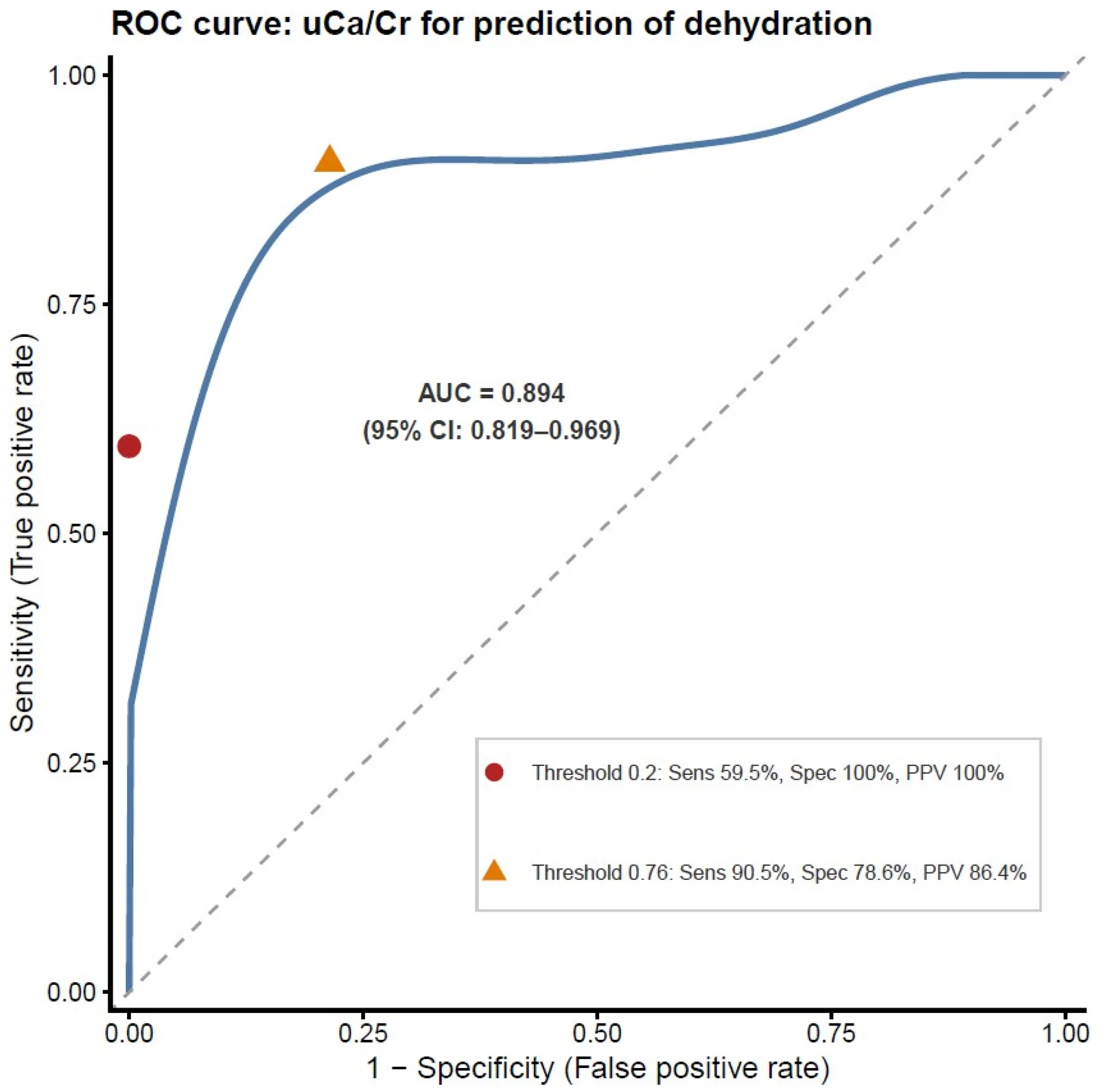
ROC curve for uCa/Cr as a predictor of dehydration (n = 70, diuretic-filtered). Red circle: threshold 0.2 mmol/mmol (sensitivity 59.5%, specificity 100%, PPV 100%). Orange triangle: Youden-optimal threshold 0.76 mmol/mmol (sensitivity 90.5%, specificity 78.6%, PPV 86.4%). AUC = 0.894 (95% CI: 0.819–0.969).

ROC analysis for uCa/Cr as a predictor of dehydration yielded an AUC of 0.894 (95% CI: 0.819–0.969; Figure 3). At the pre-specified threshold of 0.2 mmol/mmol, uCa/Cr achieved sensitivity 59.5%, specificity 100%, PPV 100%, and NPV 62.2%. The Youden-optimal threshold of 0.76 mmol/mmol provided sensitivity 90.5%, specificity 78.6%, PPV 86.4%, and NPV 84.6% (Table 2). FeCa achieved an AUC of 0.813 (95% CI: 0.714–0.912), significantly inferior to uCa/Cr (DeLong *p* = 0.012); it is therefore not presented in Figure 3.

### 3.5. Logistic Regression and CKD Sensitivity Analysis

In logistic regression, uCa/Cr was a significant independent predictor of dehydration across all three models (Table 2). In the fully adjusted model (Model 3: uCa/Cr + CKD + age + sex), uCa/Cr remained strongly associated with dehydration (OR 0.112 per unit increase, 95% CI: 0.030–0.307, *p* < 0.001). CKD was not independently associated with dehydration status after adjustment for uCa/Cr (OR 3.63, *p* = 0.191), and its addition did not significantly improve model fit (likelihood ratio test *p* = 0.184). In the non-CKD subgroup (n = 56), AUC was 0.864 (95% CI: 0.765–0.963) and uCa/Cr remained significantly lower in dehydrated patients (median 0.192 vs. 1.046 mmol/mmol, *p* < 0.001). In the CKD subgroup (n = 14), uCa/Cr also remained significantly lower in dehydrated patients despite the small sample (*p* = 0.022), with an AUC of 1.000.

### 3.6. Sex-Stratified Analysis

**Figure 4:**
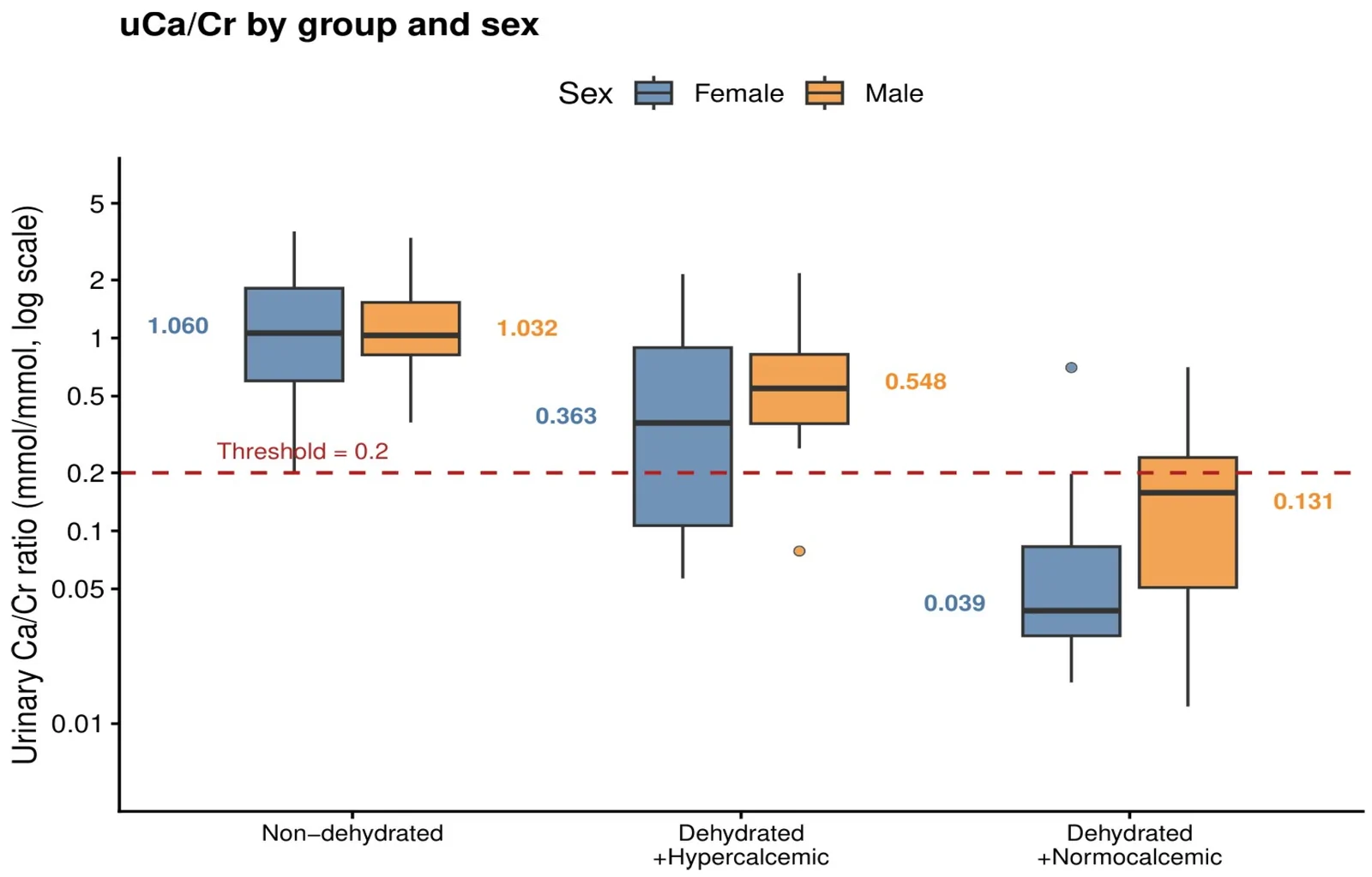
uCa/Cr by patient group and sex (n = 70, diuretic-filtered), log scale. Significant in males (*p* < 0.001; n = 17 non-dehydrated, n = 24 dehydrated) and females (*p* = 0.001; n = 11 non-dehydrated, n = 18 dehydrated).

The discriminatory ability of uCa/Cr was consistent across both sexes (Figure 4). In males (n = 41), median uCa/Cr was 1.032 mmol/mmol in non-dehydrated and 0.196 mmol/mmol in dehydrated patients (*p* < 0.001). In females (n = 29), median uCa/Cr was 1.060 and 0.094 mmol/mmol respectively (*p* = 0.001). The dehydrated + hypercalcemic group had a higher proportion of females (67%), while sex distribution was similar in the other two groups.

## 4. Discussion

This retrospective cohort study demonstrates that random uCa/Cr is a highly specific, independently informative, and clinically accessible predictor of dehydration in hospitalized patients with or without hypercalcemia. Its diagnostic performance is superior to FeCa, persists after adjustment for CKD, and is consistent across both sexes. To our knowledge, this is the first study to systematically evaluate uCa/Cr as a dehydration biomarker across a spectrum of hypercalcemic and normocalcemic presentations, with logistic regression adjustment for the principal biological confounder.

The central finding is a stepwise reduction in uCa/Cr from non-dehydrated (median 1.046) to dehydrated + hypercalcemic (0.484) to dehydrated + normocalcemic patients (0.056), with all pairwise comparisons significant. This gradient supports a continuum model in which dehydration progressively suppresses urinary calcium excretion, independently of whether hypercalcemia has yet developed. The finding that uCa/Cr is lower in normocalcemic than in hypercalcemic dehydrated patients is physiologically coherent: in the hypercalcemic group, the calcium load from hemoconcentration partially offsets tubular reabsorption, maintaining a modestly higher ratio. This stepwise pattern was mirrored by FeCa, though FeCa failed to distinguish the non-dehydrated from the dehydrated + hypercalcemic group-a clinically critical distinction that uCa/Cr achieves.

The mechanistic basis for dehydration-induced hypocalciuria involves enhanced calcium reabsorption at multiple nephron segments [15,16]. Volume depletion promotes sodium reabsorption in the proximal tubule with passive paracellular calcium co-transport via claudin proteins [15,16]. In the distal convoluted tubule, PTH-mediated transcellular calcium reabsorption via TRPV5, calbindin-D28k, NCX1, and PMCA1b further reduces urinary calcium excretion [16,17]. When dehydration is severe enough to cause hypercalcemia, a self-perpetuating cycle may ensue: sustained hypercalcemia can impair aquaporin-2 channel function in the collecting duct, causing nephrogenic diabetes insipidus, polyuria, and further volume depletion [18,19,20,21].

The 100% specificity and PPV at the 0.2 mmol/mmol threshold are the most clinically actionable findings. This threshold, previously established for thiazide-induced hypocalciuria [7] and Gitelman syndrome [8,11], appears transferable to dehydration. For screening, the Youden-optimal threshold of 0.76 mmol/mmol offers 90.5% sensitivity and 78.6% specificity. We propose a two-threshold clinical approach: uCa/Cr > 0.76 argues against dehydration as the primary cause; uCa/Cr < 0.2 confirms it. Values between 0.2 and 0.76 warrant clinical judgment.

The PTH findings provide a complementary diagnostic dimension. PTH suppression was significantly less common in dehydrated patients (24.4%) than in non-dehydrated hypercalcemic patients (51.5%, *p* = 0.018). Among non-dehydrated patients, suppressed PTH reflects the humoral or local mechanisms of hypercalcemia from malignancy and other non-PTH-mediated causes. In contrast, dehydrated patients-particularly those without hypercalcemia-had elevated PTH (median 57.2 pg/mL), consistent with secondary hyperparathyroidism driven by volume depletion. This pattern suggests that the combination of uCa/Cr < 0.2 mmol/mmol and non-suppressed PTH offers a clinically intuitive and mechanistically coherent diagnostic approach to dehydration-associated hypercalcemia.

The logistic regression analysis directly addresses concerns about CKD confounding. CKD prevalence was notably higher in the dehydrated + hypercalcemic group (54%), raising the possibility that reduced GFR rather than dehydration per se drove hypocalciuria in this subgroup [22]. However, uCa/Cr remained a highly significant independent predictor of dehydration after adjustment for CKD (OR 0.112, *p* < 0.001), and CKD itself was not independently associated with dehydration status (*p* = 0.191). The sensitivity analysis in non-CKD patients (AUC 0.864) confirms that diagnostic accuracy is not dependent on CKD being present. Notably, FeNa and FeUrea did not differ significantly across groups, likely reflecting early intravenous fluid administration before urine samples were collected; this further supports uCa/Cr as a more stable dehydration marker than the fractional excretion indices [23,24].

The relationship between CKD and urinary calcium excretion warrants specific comment. In CKD, reduced GFR decreases the filtered calcium load, while secondary hyperparathyroidism-driven by hypocalcemia, hyperphosphatemia, and vitamin D deficiency-enhances distal tubular calcium reabsorption via the PTH–TRPV5 axis, together tending to reduce uCa/Cr below levels seen in healthy individuals [25]. One might therefore expect CKD alone to produce low uCa/Cr values, potentially confounding our results. However, our data demonstrate that even within the CKD subgroup, uCa/Cr was significantly lower in dehydrated than non-dehydrated patients (*p* = 0.022), suggesting that dehydration-driven tubular calcium conservation exerts an additional effect that is detectable above the CKD baseline. This is mechanistically coherent: the proximal sodium-calcium co-transport pathway activated by volume depletion operates upstream of and independently of GFR. The practical implication is that a very low uCa/Cr in a CKD patient should still raise clinical suspicion for dehydration, rather than being attributed solely to renal dysfunction.

Comparison with conventional dehydration markers is instructive. The blood urea nitrogen (BUN)/creatinine ratio is widely used as a biochemical surrogate for dehydration, with a ratio above 20 often cited as suggestive of prerenal azotemia [23]. However, this ratio is confounded by protein intake, gastrointestinal bleeding, corticosteroid use, and muscle mass, limiting its specificity for true volume depletion. Similarly, FeNa and FeUrea, commonly used to distinguish prerenal from intrinsic renal failure, were not significantly different across our three groups, likely because urine samples were collected after emergency department fluid administration had partially normalized sodium handling [23,24]. In contrast, uCa/Cr appears to be more resistant to early fluid correction, potentially because the tubular calcium conservation response to volume depletion is more sustained. These observations suggest that uCa/Cr may be a more specific and robust dehydration marker than conventional indices, though prospective head-to-head comparisons in larger cohorts are needed to confirm this.

The higher CKD prevalence in the dehydrated + hypercalcemic group likely reflects a biological interaction: impaired GFR reduces the filtered calcium load and promotes tubular calcium retention, lowering the threshold at which dehydration triggers frank hypercalcemia [22]. Similarly, the predominance of females in the dehydrated + hypercalcemic group (67%) may reflect known sex differences in calcium metabolism and PTH responsiveness, with estradiol influencing serum calcium homeostasis in postmenopausal women [26]. The sex-stratified analysis confirmed that uCa/Cr discriminates dehydration equally well in both sexes.

Several limitations should be acknowledged. This is a single-center retrospective study with a modest sample size of 70 for urinary analyses, limiting the power of subgroup analyses. There is no universally accepted gold standard for dehydration diagnosis; our combined clinical-biochemical definition, while systematic and internally validated, is necessarily composite. Urine samples were collected at admission, and some patients may have received modest fluid volumes in the emergency department before ward admission, which could partially attenuate uCa/Cr values. However, this would bias results toward the null, making our findings conservative. Bioimpedance analysis, which provides an objective measure of volume status, was not performed and represents a recognized limitation in the objective quantification of volume depletion. Full acid–base parameters (pH, bicarbonate) were not systematically collected, precluding pH correction of ionized calcium measurements. Urine albumin-to-creatinine ratio and protein-to-creatinine ratio were not collected as part of this study’s protocol. Chronic medication data were limited to diuretic use; SGLT2 inhibitor use, which can promote dehydration through glucosuria-driven osmotic diuresis, was not systematically recorded and cannot be excluded as a potential confounder. No patients with autosomal dominant polycystic kidney disease (ADPKD) were identified in this cohort. We lacked baseline creatinine data to formally define acute kidney injury, and PTH data were missing in 18.8% of patients. Future prospective multi-center studies with standardized urine collection protocols, larger samples, and external validation cohorts are needed to validate these thresholds and confirm the clinical utility of combining uCa/Cr with PTH.

## 5. Conclusions

Random uCa/Cr is a reliable, non-invasive, and highly specific predictor of dehydration in patients presenting with hypercalcemia or volume depletion. Its diagnostic performance is superior to FeCa and is preserved across CKD and non-CKD subgroups and both sexes. A uCa/Cr below 0.2 mmol/mmol, particularly in the context of a non-suppressed PTH, provides strong support for dehydration as the primary etiology of hypercalcemia. These findings support the routine inclusion of urine calcium and creatinine in the initial biochemical evaluation of hypercalcemia in hospitalized patients.

## Figures and Tables

**Table 1:** Patient characteristics by group.

Variable	Non-Dehydrated (n = 37)	Dehydrated + Hypercalcemic (n = 24)	Dehydrated + Normocalcemic (n = 35)	*p* Value
n in urinary analyses	28	15	27	—
***Serum variables—median (IQR)***				
Age (years)	70.0 (59.0–83.0)	82.0 (74.8–90.2)	78.0 (56.0–85.0)	0.060
Plasma sodium (mEq/L)	137.0 (134.0–140.0)	139.5 (136.0–145.5)	143.0 (137.0–154.5)	0.002
Urea (mg/dL)	49.0 (34.0–82.5)	95.0 (61.0–166.5)	117.0 (65.5–151.5)	<0.001
Creatinine (mg/dL)	0.9 (0.7–1.4)	1.8 (1.2–3.6)	1.7 (1.1–2.7)	<0.001
Albumin (g/dL)	3.6 (3.0–4.0)	3.7 (3.1–4.1)	3.1 (2.6–4.0)	0.301
Total protein (g/dL)	6.5 (6.1–7.2)	6.8 (6.2–7.6)	6.5 (6.1–7.2)	0.519
Total calcium (mg/dL)	12.3 (11.0–13.6)	11.7 (10.7–13.1)	8.8 (8.2–9.6)	<0.001
Corrected calcium (mg/dL)	12.6 (11.7–13.8)	12.3 (10.9–13.4)	9.7 (9.1–9.9)	<0.001
Ionized calcium (mmol/L)	1.5 (1.5–1.7)	1.5 (1.4–1.6)	1.2 (1.1–1.2)	<0.001
PTH (pg/mL)	18.2 (0.0–105.0)	22.5 (7.1–56.5)	57.2 (40.5–92.0)	0.078
***Urine variables—median (IQR) ^a^***				
Urine osmolality (mOsm/kg)	412.5 (263.2–528.8)	515.0 (374.5–585.0)	531.0 (439.0–729.8)	0.048
Urine sodium (mEq/L)	48.0 (22.0–75.0)	40.0 (27.5–66.0)	45.0 (24.0–79.0)	0.971
Urine creatinine (mg/dL)	40.7 (22.8–77.5)	92.8 (67.2–101.2)	83.6 (60.4–109.3)	0.009
uCa/Cr (mmol/mmol)	1.0 (0.8–1.8)	0.5 (0.2–0.9)	0.1 (0.0–0.2)	<0.001
FeCa (%)	3.3 (1.9–5.7)	2.0 (1.6–3.8)	0.5 (0.2–1.3)	<0.001
FeNa (%)	0.8 (0.3–2.1)	0.6 (0.3–1.0)	0.7 (0.2–1.1)	0.634
FeUrea (%)	42.9 (32.2–47.7)	32.4 (25.2–40.3)	33.9 (24.0–45.8)	0.170
***Categorical variables—n (%)***				
Male sex	22 (59.5%)	8 (33.3%)	22 (62.9%)	0.061
CKD	5 (13.5%)	13 (54.2%)	9 (25.7%)	0.001
HTN	21 (56.8%)	19 (79.2%)	19 (54.3%)	0.132
DM	16 (43.2%)	12 (50.0%)	18 (51.4%)	0.831
Furosemide	6 (16.2%)	9 (37.5%)	6 (17.1%)	0.106

Data presented as median (IQR) or n (%). *p* values from Kruskal–Wallis test (continuous) or chi-square test (categorical). ^a^ Urinary variables calculated in patients not receiving chronic diuretic therapy (n = 75); n per group reflects available urine data. Abbreviations: CKD = chronic kidney disease; DM = diabetes mellitus; FeCa = fractional excretion of calcium; FeNa = fractional excretion of sodium; FeUrea = fractional excretion of urea; HTN = hypertension; IQR = interquartile range; PTH = parathyroid hormone; uCa/Cr = urinary calcium-to-creatinine ratio.

**Table 2:** Diagnostic performance of uCa/Cr and FeCa.

**A. Urinary markers by group (diuretic-filtered, n = 70)**					
Group	n	uCa/Cr—median (IQR) (mmol/mmol)		FeCa—median (IQR) (%)	
Non-dehydrated	28	1.046 (0.780–1.754)		3.26 (1.87–5.69)	
Dehydrated + Hypercalcemic	15	0.484 (0.189–0.927)		2.04 (1.64–3.80)	
Dehydrated + Normocalcemic	27	0.056 (0.037–0.197)		0.52 (0.20–1.26)	
**B. ROC diagnostic performance**					
Threshold	Sensitivity	Specificity	PPV	NPV	AUC (95% CI)
uCa/Cr 0.2 mmol/mmol (high specificity)	59.5%	100%	100%	62.2%	0.894 (0.819–0.969)
uCa/Cr 0.76 mmol/mmol (Youden-optimal)	90.5%	78.6%	86.4%	84.6%	—
FeCa 1.9% (Youden-optimal)	74.4%	75.0%	82.1%	65.6%	0.813 (0.714–0.912)
**C. Logistic regression—uCa/Cr as independent predictor of dehydration**					
Variable	OR	95% CI lower		95% CI upper	*p* value
*Model 1: uCa/Cr alone*					
uCa/Cr	0.109	0.029		0.297	<0.001
*Model 2: uCa/Cr + CKD*					
uCa/Cr	0.124	0.034		0.329	<0.001
CKD	3.366	0.591		33.921	0.221
*Model 3: uCa/Cr + CKD + Age + Sex (fully adjusted)*					
uCa/Cr	0.112	0.030		0.307	<0.001
CKD	3.631	0.634		35.239	0.191
Age	0.982	0.944		1.016	0.327
Sex (male)	0.943	0.257		3.384	0.928

(**A**). Kruskal–Wallis *p* < 0.001 for both markers. Pairwise comparisons after Bonferroni correction—uCa/Cr: non-dehydrated vs. dehydrated + hypercalcemic *p* = 0.017; non-dehydrated vs. dehydrated + normocalcemic *p* < 0.001; dehydrated + hypercalcemic vs. dehydrated + normocalcemic *p* = 0.001. FeCa: non-dehydrated vs. dehydrated + hypercalcemic *p* = 0.412 (ns); non-dehydrated vs. dehydrated + normocalcemic *p* < 0.001; dehydrated + hypercalcemic vs. dehydrated + normocalcemic *p* = 0.001. Hypocalciuria (uCa/Cr < 0.2 mmol/mmol): 0/28 (0%) non-dehydrated; 25/42 (59.5%) dehydrated (*p* < 0.001). Spearman correlation between uCa/Cr and FeCa: ρ = 0.883, *p* < 0.001. (**B**). DeLong test for AUC comparison (uCa/Cr vs. FeCa): *p* = 0.012. AUC = area under the curve; NPV = negative predictive value; PPV = positive predictive value. (**C**). Dependent variable: dehydration (binary). OR = odds ratio per unit increase in uCa/Cr. Likelihood ratio test Model 1 vs. Model 2: *p* = 0.184 (CKD does not significantly improve fit). CKD sensitivity analysis: AUC in non-CKD patients (n = 56) = 0.864 (95% CI: 0.765–0.963); AUC in CKD patients (n = 14) = 1.000 (*p* = 0.022).

## Data Availability

The data presented in this study are available on request from the corresponding author due to ethical and privacy restrictions.
